# Outcomes in the offspring of mothers with pre-diabetes during pregnancy: a protocol for a systematic review

**DOI:** 10.1186/s13643-015-0051-1

**Published:** 2015-05-10

**Authors:** Rukia Swaleh, Ling Zeng, Lawrence Mbuagbaw, Katherine M Morrison

**Affiliations:** Faculty of Medicine, University of Toronto, Toronto, Ontario Canada; Faculty of Health Sciences, McMaster University, Hamilton, Ontario Canada; Department of Clinical Epidemiology & Biostatistics, McMaster University, Hamilton, Ontario Canada; Biostatistics Unit, Father Sean O’Sullivan Research Centre, St Joseph’s Healthcare, Hamilton, Ontario Canada; Department of Pediatrics, McMaster University, HSC 3A59, 1280 Main St W, Hamilton, Ontario L8N 3Z5 Canada

**Keywords:** Pre-diabetes, Impaired fasting glucose, Glucose intolerance, Hyperglycemia, Offspring outcome

## Abstract

**Background:**

Despite the increasing prevalence of pre-diabetes worldwide, there is insufficient literature on the impact of gestational pre-diabetes on offspring outcomes. The objective of this systematic review is to determine the risk of developing adverse outcomes for the offspring in women with pre-diabetes compared to women with normal glucose levels and women with gestational diabetes mellitus.

**Methods/design:**

A systematic search of the published literature will be conducted for experimental and observational studies that report outcomes in the offspring of mothers with pre-diabetes during pregnancy. Databases including EMBASE, PsycINFO, and PubMed will be searched from 1979 (the year when the terms impaired glucose tolerance and pre-diabetes were coined) to December 2014. Screening of identified articles and data extraction will be conducted in duplicate and independently. Methodological quality of the included studies will be assessed using the Newcastle-Ottawa scale. Discrepancies will be resolved by consensus or by consulting a third author. Meta-analyses will be performed, and findings will be reported according to the preferred reporting items for systematic reviews and meta-analyses (PRISMA) and the meta-analysis of observational studies in epidemiology (MOOSE) guidelines.

**Discussion:**

Determining the effect of pre-diabetes on offspring outcome will be important for clinicians providing care to pregnant women and their offspring. This review will also identify any gaps in the current literature on this topic and provide direction for future research in this area of study.

**Systematic review registration:**

PROSPERO CRD42015015536

## Background

The prevalence of pre-diabetes (a condition in which blood sugar levels are higher than normal but are below levels found in clinical diabetes) is rising worldwide. It is estimated that about 470 million people will be diagnosed with pre-diabetes globally by the year 2030 [1]. Pre-diabetes includes impaired fasting glucose, impaired glucose tolerance, and hemoglobin A1c of 6% to 6.4% [2]. Currently, the crude prevalence of pre-diabetes in the United States is roughly 30% among individuals greater than 20 years old [3]. The Canadian Diabetes Association (CDA) estimates that 5.7 million out of the 35 million Canadians have pre-diabetes [4]. Adults with pre-diabetes have an increased risk of developing Type 2 diabetes mellitus (T2DM) [5] and cardiovascular disease [6].

During pregnancy, women can develop a dysglycemic state known as gestational diabetes mellitus (GDM). Although most women with GDM have normal blood sugars after delivery, the risk of developing subsequent T2DM is higher [7]. The diagnostic criteria for GDM vary internationally but generally involve a two-step process: an initial screening challenge with glucose followed by a standardized 75 (or 100) g oral glucose tolerance test (OGTT) if the screen is abnormal. More details on the classification of GDM are included in the methods. This dysglycemic state during pregnancy has been found to influence fetal development and has both early and long-term implications for offspring health [8]. GDM is independently associated with macrosomia, shoulder dystocia, pre-term delivery, caesarian deliveries, and perinatal mortality [9]. Long-term outcomes of GDM in the offspring include obesity and abnormal glucose metabolism during childhood, adolescence, and adulthood [10,11]. Recent results from the hyperglycemia and adverse pregnancy outcome (HAPO) study have suggested that even milder levels of hyperglycemia can have adverse effects on offspring outcome [12]. Thus far, it is clear that research has focused on the effects of GDM on the fetus. Despite the rising prevalence of pre-diabetes, studies on the impact of gestational pre-diabetes on offspring outcomes are still scarce [11].

In this study, we will conduct a systematic review of the existing literature to identify studies that address both short- and long-term outcomes in the offspring of pregnant women with pre-diabetes. Our objective is to determine the risk of developing adverse outcomes for the offspring in women with pre-diabetes compared to women with normal glucose levels and women with GDM. We will compare the three groups and report two risk comparisons: (1) pre-diabetes *vs*. normal glucose levels and (2) pre-diabetes *vs*. GDM. We hypothesize that offspring with intrauterine exposure to pre-diabetes would have a higher risk of developing adverse outcomes compared to those without the exposure. When compared to offspring exposed to GDM, we hypothesize that those with intrauterine exposure to pre-diabetes would have a lower risk of adverse outcomes. Lastly, we aim to identify any gaps in the literature that exist on this topic.

## Methods/design

### Criteria for study inclusion

#### Types of studies

Experimental (randomized and nonrandomized) and observational studies (prospective/retrospective cohort and case control) will be considered. Articles must be in English and address offspring outcomes of mothers with pre-diabetes during pregnancy. Review articles and articles without a pre-diabetic study group or without a control group will be excluded.

#### Types of participants

We will include studies with pregnant women. There are three groups of interests: (1) those with pre-diabetes/abnormal glucose tolerance/impaired fasting glucose during pregnancy, (2) those with normal glucose levels, and (3) those with diagnosed gestational diabetes. We will compare the mode of delivery and the risk of adverse outcomes in offspring across the three groups. Risk comparisons will be made between group (1) and group (2), as well as group (1) and group (3).

#### Exposure

During pregnancy, pre-diabetes is diagnosed using a two-step approach – Glucose Challenge Test (GCT) followed by an OGTT. Various cutoffs for the GCT and OGTT are used during diagnosis and are based on different criteria such as the National Diabetes Data Group (NDDG), Carpenter and Coustan (C&C), and World Health Organization (WHO) criteria [13,14]. See Table 1 for plasma glucose values used by the different diagnostic criteria. Other criteria for pre-diabetes will also be included if they are clearly defined. A conversion table for blood glucose monitoring will be used to convert units between mg/dL and mmol/L [15].

**Table 1:** **Plasma glucose values used as diagnostic criteria for gestational diabetes mellitus and pre-diabetes**

	**NDDG (1979)** ^**a**^	**C&C (1982)** ^**a**^	**WHO (2006)** ^**b**^
1 hr-GCT (50 g) at 24 to 28 weeks gestation	≥140 (7.8)	≥140 (7.8)	92 to 125 (5.1 to 6.9)
3 hr-OGTT			
1 hr	≥190	≥180	≥180 (10)
2 hr	≥165	≥155	153 to 199 (8.5 to 11)
3 hr	≥145	≥140	

Values are expressed in mg/dL (mmol/L). ^a^100 g OGTT; ^b^75 g OGTT. Gestational pre-diabetes is confirmed when there is an abnormal result in the GCT test and either normal or 1 abnormal OGTT test result [23,24]. If there are two or more abnormal values in the OGTT, the patient is diagnosed with GDM [14]. C&C, Carpenter and Coustan; GCT, glucose challenge test; NDDG, National Diabetes Data Group; OGTT, oral glucose tolerance test; WHO, World Health Organization.

#### Outcomes

Our primary outcomes of interest will be:

- Birth weight (kg) and offspring weight/body mass index (BMI) in childhood. The childhood data will be categorized as neonatal (0 to 28 days), infant (0 to 1 year), toddlers (1 to 2 years), and preschool (3 to 5 years). For studies with multiple time points, data corresponding to the oldest age within each category will be used.

Our secondary outcomes of interest will be:

- Macrosomia (a birth weight above 4 kg or above the 90th percentile).
- Prematurity (number of offspring born prior to 37 weeks gestation).
- Emergency cesarean sections (number of women who deliver by C-section).
- Mean Apgar scores at 1 min and at 5 min, and
- Offspring glycemic status (% of offspring with glucose intolerance).

Outcomes will be reported according to three categories: pregnancy complications, neonatal outcomes, and long-term offspring outcomes. Data on pregnancy complications will include prematurity and emergency cesarean sections. Neonatal outcomes will include mean birth weight, proportion of children with macrosomia, and mean Apgar scores at 1 min and at 5 min. In the event that birth weight is reported in other units such as grams or pounds, a unit conversion will be conducted. Long-term offspring outcomes will include offspring BMI and percent of offspring with glucose intolerance.

### Search strategy for identification of studies

We will conduct a search of the published literature looking for studies reporting outcomes in the offspring of mothers with pre-diabetes during pregnancy.

#### Electronic searches

We will search the following databases from 1979, which is the year when the term impaired glucose tolerance and pre-diabetes were coined [16]: EMBASE, PsycINFO, and PubMed. The following subject headings (MeSH) and key terms will be used in various combinations and adapted for each database: *pre-diabetes, prediabetes, impaired fasting glucose, impaired glucose tolerance, glucose intolerance, dysglycemia, hyperglycemia, pregnancy, maternal, mother, offspring, outcomes, impact, consequences, metabolic, metabolic syndrome, obesity, cholesterol, blood pressure, hypertension, atherosclerosis, cardiovascular.* The following section summarizes our proposed search strategy on Ovid MEDLINE®:

#### Proposed search strategy

Database: Ovid MEDLINE®

1. pre-diabetes.mp.
2. prediabetes.mp.
3. impaired fasting glucose.mp.
4. impaired glucose tolerance.mp.
5. glucose intolerance.mp.
6. dysglycemia.mp.
7. hyperglycemia.mp.
8. pregnancy.mp.
9. maternal.mp.
10. mother.mp.
11. offspring.mp.
12. outcomes.mp.
13. impact.mp.
14. consequences.mp.
15. metabolic.mp.
16. metabolic syndrome.mp.
17. obesity.mp.
18. cholesterol.mp.
19. blood pressure.mp.
20. hypertension.mp.
21. atherosclerosis.mp.
22. cardiovascular.mp.
23. 1 or 2 or 3 or 4 or 5 or 6 or 7
24. 8 or 9 or 10
25. 12 or 13 or 14 or 15 or 16 or 17 or 18 or 19 or 20 or 21 or 22
26. 11 and 23 and 24 and 25
27. glucose level.mp.
28. 23 or 27
29. 11 and 24 and 25 and 28
30. abnormal glucose test.mp.
31. 28 or 30
32. 11 and 24 and 25 and 31
33. remove duplicates from 32
34. 33 and “human”.sa_suba

#### Reference lists

We will search the reference lists of relevant citations for articles of interest.

#### Grey literature

We will contact authors and experts in the field for any relevant material.

### Data collection and analysis

#### Screening

Two reviewers (RS and LZ) will independently select studies that meet the following criteria: any article published in the English literature addressing outcomes in offspring of mothers with pre-diabetes during pregnancy. Citations and abstracts will be screened for relevance, and duplicate citations will be excluded. Full text screening will be conducted and article eligibility will be evaluated using a standardized and pre-tested form. Disagreements will be resolved by consensus, with consultation of a third investigator (KM) when resolution cannot be achieved. Corresponding authors will be contacted in the event that the publication (1) does not permit us to decide its eligibility, (2) is unclear and may be subject to multiple interpretations, or (3) has collected data but did not report data that are relevant to our study analysis.

#### Data extraction

Two reviewers (RS and LZ) will independently extract data in duplicate from included studies, using a standardized and pre-tested data extraction form. Data will include the study design, setting, sample size, quality score, how glucose intolerance was measured, confounders, and details of the outcomes. Specifically, outcomes will be categorized into mode of delivery, neonatal outcomes, and long-term offspring outcomes. Rates of study and comparison groups, along with z-scores, odds ratios, *P* values, and confidence intervals, will be recorded. If offspring weight/BMI data are reported as z-scores, authors will be contacted in order to obtain the raw data.

#### Assessment of methodological quality

Two reviewers (RS and LZ) will independently score the quality of included studies using the Newcastle-Ottawa scale (NOS) [17]. The NOS assesses nonrandomized/observational studies based on eight items categorized into three groups: (1) the selection of the study groups, (2) the comparability of the groups, and (3) the ascertainment of either the exposure or outcome of interest [17]. Discrepancies will be resolved by consensus or by consulting the third investigator (KM).

Agreement on screening, data abstraction, and methodological quality will be measured using the Kappa statistic [18].

### Analysis and reporting

For outcomes reported as rates or proportions, we will calculate a pooled estimate of the proportion by weighting the studies by their respective sample sizes. We will use the random effects model in the meta-analysis of proportions and investigate sources of heterogeneity when inconsistency is high (*I*^2^ > 75%) [19]. For similar continuous outcomes with the same measurement scales, we will report the mean difference (SD). If measurement scales are different or not readily convertible, we will report the standardized mean difference. Comparisons of pooled estimates will be made between pre-diabetic/diabetic groups and the nondiabetic groups of pregnant women. We will analyze the data using Review Manager (RevMan) V.5.3 (The Nordic Cochrane Centre, The Cochrane Collaboration, 2014, Copenhagen, Denmark) and Statistical Analysis Software (SAS) V.9.3 (SAS Institute, 2009, Cary, NC, USA) [19]. We will report our findings according to the preferred reporting items for systematic reviews and meta-analyses (PRISMA) and the meta-analysis of observational studies in epidemiology (MOOSE) guidelines [20,21]. When statistical data pooling does not yield meaningful results, such as in the presence of considerable clinical heterogeneity or irreconcilable outcome measures, we will conduct a narrative synthesis. We will construct a funnel plot and check for asymmetry as well as run a meta-regression test for publication bias [22].

#### Subgroup analysis

We will perform a primary subgroup analysis based on maternal BMI, study design (experimental *vs*. observational), and methodological quality. Subgroup effect sizes from the meta-analysis will be reported in comparison to the overall effect size. Any subgroup differences identified in the primary subgroup analysis will be described, and our findings will be interpreted in the light of these differences. Further exploratory analysis will be conducted on the following factors if they have been reported at study level: gestational age, prematurity, maternal age, ethnicity, socioeconomic status, and level of maternal antenatal care.

## Discussion

The effects of gestational diabetes mellitus on offspring outcomes, both short term and long term, have been clearly documented, unlike the effects of pre-diabetes. In this review, we intend to determine the impact of lesser degrees of glucose intolerance (pre-diabetes) during pregnancy on offspring health. We will document the risk of adverse outcomes in the offspring of women with gestational pre-diabetes as compared to women with normal glucose tolerance and those with GDM. Determining the effect of pre-diabetes on offspring outcome will be important for clinicians providing care to pregnant women. If pre-diabetes is found to have adverse effects, this may influence clinical practice by increasing the number of women screened and treated for glucose intolerance during pregnancy. Furthermore, if long-term consequences are identified, these offspring may benefit from close follow-up and attention-to-lifestyle behaviors to prevent long-term cardiometabolic disorders as is currently recommended for offspring of mothers with GDM. This review will also identify any gaps in the current literature on this topic and provide direction for future research in this area of study.

## Abbreviations

BMI: body mass index
GCT: glucose challenge test
GDM: gestational diabetes mellitus
HDLc: high-density lipoprotein levels
LDLc: low-density lipoprotein levels
NOS: Newcastle-Ottawa scale
OGTT: oral glucose tolerance test
SD: standard deviation
T2DM: type 2 diabetes mellitus
